# Abstract of the Proceedings of the Buffalo Medical Association

**Published:** 1857-02

**Authors:** Sanford B. Hunt


### ART. IV.—*Abstract of the Proceedings of the Buffalo Medical Association.*

THURSDAY EVENING, Jan. 6, 1857.

The association met.

Present—The President, Dr. Eastman, in the chair. Drs. Hawley, Wyckoff, Lemon, Newman, Wilcox, Nott, White, Nichols, Rochester, Strong, Hunt, Hutchins, and Flint. Also, Drs. Ham and Van Pelt, of Williamsville, honorary members, and Dr. Rogers, of Buffalo, invited guest.

The minutes of the preceding meeting were read and approved.

Dr. Augustus Jeyte was elected to membership.

*Dr. P. H. Strong*, from the committee on "Fistula in Ano in its relations to Pulmonary Consumption," then read the following report:

Your committee, appointed some months since to report at "his convenience, on Fistula in Ano in its relations to Pulmonary Tuberculosis," would ask leave to report:

That whilst the subject is one of very considerable pathological and therapeutical interest, he has not been able, after not a little examination of authorities, both of formal treatises upon practical medicine and monographs, to find that the subject has attracted the degree and kind of attention that it seems to deserve. Not that the *fact* of the coëxistence of the two affections is wholly unnoticed. The phenomenon has not only been observed, but written of, and has been influencing practice for many years, but almost always regarded as an incidental or accidental complication of no especial importance or signification. Hence there is nearly or quite a total lack of cases recorded in which the complication of anal fistula and tubercle has been not only noticed, but its history carefully and statedly traced and its bearings and relations critically analyzed.

It follows that in so far as the resolution (under which your committee was appointed) contemplated a report upon the bibliography and natural history of the subject, your committee is reluctantly compelled to make the return of "*non est inventus;*" nothing but the vaguest and most casual allusion to the fact of the coëxistence of the two affections, having yet appeared to your committee.

The subject seems to have been consigned to that vast limbo of vanity — that huge receptacle, into which in all ages of medicine so many *supposed* isolated unrelated *dumb* facts, have been so unceremoniously pitched — there to remain until a later age has wrought out their true significance and import, indicated their true relations to other facts, and given them their true position in that temple of truth, which the votaries of science, in all departments, have erected or are erecting.

That fistula in ano is a frequent concomitant of pulmonary tuberculosis, has been sufficiently recognized. But whether it acts any part in its etiology, pathology or treatment, or is a mere accident, seems to be yet undetermined. That it has *some* relation seems potent enough; but whether of cause or effect, or if, indeed it be anything more than mere coincidence, has not, to my knowledge, been well settled.

By some it is regarded, apparently, as is a quarrelsome neighbor — rarely to be alluded to, and never to be meddled with, in a word — to be let alone *severely;* by others as a kindly helpmate, a welcome adjuvant, a cherished ally in the work of either holding at bay a too formidable enemy, or of deferring yet a little, a dreaded but unavoidable capitulation. But howsoever regarded there seems to be a plentiful lack of cases accurately observed and well recorded, upon which to base a satisfactory generalization and estimate of the relations of the two affections.

From this cause, and from the fact that I have personal observations to present illustrative of the subject, so far as the written and what may be termed the natural history of the subject in question is contemplated in the society's resolution, your committee would appear to be exonerated from farther duty in this regard.

But there *is* a phase of the subject which to your committee seems obviously to demand, and very likely to *reward* due investigation. I refer to fistula in ano, in what may be termed its therapeutical bearings upon tuberculosis. Here, again, the records of medicine furnish no well-defined trustworthy results, worthy of mention. What is known, or rather believed, seems to rest not upon any accurate analysis of a series of cases, but upon a vague tradition which has been handed down, in unquestioning confidence, from one generation to another, and that, too, with a force not diminishing like the law of gravitation, (which would have been more rational as well as natural) but increasing "as the space of the distance increases," from its source.

The sentiment to which especial reference is made, and to which I would for a few minutes limit myself and call the attention of the association, would, perhaps, find nearly a correct expression in this formula, viz:

*That the relations of fistula in ano to pulmonary tuberculosis are such, that its presence either as antecedent to, or coëxistent with, the latter, is a desideratum, and to be sedulously cherished, having reference to its (pulmonary tuberculosis) prevention in the one case, and to its cure or favorable modification in the other.*

In searching for the origin of this sentiment, your committee has not been able to discover its legitimate parentage. It seems to be multiparous, and like many of its congeners in the opinions and views of early medicine, it appears also to be one of the "traditions of the elders," the very antiquity and mistiness of which it may be, furnish about all its claim for credence and currency. It is scattered all along the track of authorship, and enshrined as a precious and sacred relique in the creed of practice of practitioners of our art in every age. So deeply is it imbedded in the convictions of medical men of the past and of the present age, that to modestly suggest a doubt as the merit of its claim to belief, is well nigh regarded as an act, if not of profanity, at least of temerity.

Notwithstanding this, your committee has ventured long since to entertain a doubt as to the correctness of this view, at least without some material modification or limitation, and with your leave he will now venture to give expression to those doubts, with some of the reasons therefor, in the hope of having them met and laid on the one hand, or culminate into a juster theory and practice on the other, by the maturer visions and observations of the gentlemen of the association.

I now arraign this dogma before this bar and challenge it to show cause why its claim for further favorable regard should not be discarded. And shall proceed to indicate some of the reasons which, to my own mind, are sufficient to sustain a verdict of condemnation.

*First.* If it had ever any rationale, it was based upon a thoroughly exploded pathology of the disease (pulmonary tuberculosis) which it was thought to avert, or control.

If one who favors the course under discussion, is asked upon what principle the maintaining of anal fistula is relied upon in this disease, the answer, for the most part, is as a derivation or revellant—a class of remedies, which are never intelligently given, in your committee's judgment, except in inflammatory and congestive affections. I believe that no fact in pathology rests upon more enlightened and truthful observations than that tuberculosis consists inherently of neither the one nor the other. Hyperamia may, perhaps, precipitate the development, and complicate and, perhaps, aggravate tuberculosis; but it is now a well settled fact, I believe, that it is an accidental, and not an essential element of the disease, though Louis contends that neither bronchitis, nor pneumonia, nor pleuritis, have any effect in exciting pulmonary tuberculosis. When existing, even the hyperamia may be wholly abated by appropriate means, and yet the disposition of tubercle proceed unchecked and unmodified. Being essentially a disease of nutrition it follows that any means that are not addressed to the correction of the assimilative and nutritive functions, can at the best be only innocuous: consisting proximately of a deficiency of certain elements, and a substitution of certain other elements in the blood, all consequent upon and attended by a depreciated and diminished vitality, what can be more plainly indicated than the assiduous husbanding of the remaining vital force, and adding thereto by every possible eligible means? But when we add the conceded fact that any suppurative or ulcerative action is measurably both a cause and proof of impaired vital energy, how well nigh preposterous is such a reliance in the case before us. To remove any hyperæmic complication even of tubercular disease, there are more rational and far more reliable and well recognized resources at hand.

But in combating this formidable disease, enlightened pathology and etiology point imperiously to far other instrumentalities—means that look both to restoring and maintaining intact the animal and vital functions, as well as to eliminating the tubercular dyscrasy which constitute the essence of the disease. Aught less than this must be vain, and aught opposed worse than vain.

The treatment by derivants or revellants, and other reducing means being suggested to meet indications that later observations have proved not to exist, and precisely the contrary *to* exist, what can be more rational than that with the change of view of the nature of the affection, there should be a corresponding change of its requirements. Consistency seems to demand that we either recede from our present estimate of the essential nature of tuberculosis, or else recede from a treatment predicated upon a wholly erroneous theory of it, more especially as such treatment has not the negative merit of being not indicated, but is positively contra-indicated. The propriety of abating fistula seems thus affirmed by the received etiology, pathology, and, for the most part, the therapia of tuberculosis.

It is pertinent to remark here, that these suggestions are intended to apply here both to the case of anal fistula when antecedent to pulmonary tuberculosis, it is relied on as a curative agent in its *early and arrestable stages*. Their application should be farther limited, too, to the fistula and tuberculosis, considered as *disjuncti morbi*, or when the ulcerative product is essentially the pus globule, and not of tubercular character.

The consideration of the course that is expedient at the later stage, or at the full and extensive development of tuberculization, as also what is required in the case when the discharge of the fistula is tubercular, is purposely deferred to a later stage of the argument.

I now pass on to advert briefly to the second reason why I would discard the prevailing practice. It may be called, perhaps, the argument from analogy. I allude to what is conceded in reference to the treatment in what are, in a sense, analogous of tuberculosis, schirrhus and melanosis. They, like tubercle, are heterologous in character, having nothing analogous in the human system, whether the result of natural or morbid process. They are alike in depending upon a blood dyscrasy which is peculiar to each, though having points of resemblance. They are alike in having not only no normal sympathies or affinities with any of the organs or tissues of the system, but alike also in having no coalescing or blending relations with any other disease or diseased product.

Unendowed with, and insusceptible of vitality *per se*, they are only potent to destroy the vitality of the tissues wherein they are deposited. Consisting of a blood dyscrasy consequent upon some lesion of nutrition, and being non-hyperemic in their origin and essence, they (schirrhus and melanosis) thus bear a strong analogy to tuberculosis. But who has ever recommended either of them to be treated by counter-irritants, or by revellant or derivant discharges? When was it even thought expedient to institute an issue or seton, or cherish and perpetuate what is equivalent, an anal fistula, in the hope of forestalling when anticipated, or of favorably modifying when actually existing, in either of these? And yet so analogous are they to tubercle in their sources, and mode of development in their incidents, accidents, and concomitants, that it would seem to be obvious that the same general principles of treatment should obtain in their case as in that of tubercle. Would it be too much to say, that for one to seriously propose anal fistula to avert or to cure, schirrhus, (for example) would well nigh be regarded as entitling him to be thought of as an early candidate for a straight waistcoat and lunatic asylum.

Well, if wholly inadmissible for schirrhus, analogy would thus seem to indicate and demand its being discarded for tuberculosis.

Pass we now to some considerations which are operative with some minds, in holding them to the practice which is the subject of remark, viz., the non-abatement of anal fistula in anticipated, as well as in existing, though recent, tuberculosis.

We hear the remark not infrequently, from men who are able to give a reason for the faith that is in them in the treatment of other diseases, that "Theory and rationale apart they are persuaded that extinction of the fistula in such a case is perilous and unjustifiable—and reference, perchance, is made to a case, or to cases under their own observation, in which, upon the spontaneous or, perhaps,instrumental abatement of fistula, depositions of pulmonary tubercle have occurred, or being deposited, have gone on from latent tubercle to softening and death.

I submit that these cases prove nothing for the case for which they are produced, for the reason, primarily, that it is not sufficiently proven that the same result would not have transpired even with the existence of the fistula. Secondly, that even on the supposition of the favorable effects of the active fistula it may have been by abating some hyperemic accident and complication of tuberculosis, to do which there are much more efficacious and easily managed, and, therefore, rational means recognized.

*Thirdly.* The treatment under discussion was originated when far different and demonstrably erroneous views prevailed regarding the cause, the nature, and by consequence the requirements of tubercular disease. The treatment was then a logical emanation from those defective views. But we have now taken a long stride forward in knowledge of the cause and the essence of the disease; a stride which necessitates a very different, almost wholly opposed plan of management. But the cases supposed, to which we adverted, while they fail to demonstrate the good results of continuing, and the ill results of abating the fistula: yet *do* go far to demonstrate the evil results of the non-adoption of what every well instituted mind now regards as a *sine qua non* for the successful treatment of pulmonary tuberculosis, viz., the most positive tonic and stimulant course, the most generous diet and regimen, with the freest outdoor exposure and exercise, that is possible. To trust to the course discussed, at this late day, seems to be the twofold offence of failure to do what we clearly and most efficiently ought to have done, superadded to doing that which we positively ought not to have done.

For these reasons, among others, the cases supposed are wholly inadequate to establish the merit of the prevalent practice. The abatement of the fistula is thus, inferentially at least, the first indispensable, *albeit relatively slight* step toward fitting the therapia of tuberculosis for creditable association with her measurably well established and well favored sisters—etiology, pathology, and diagnois.

If the views your committee have thus imperfectly presented be correct, they sustain a verdict of condemnation against the practice of cherishing and maintaining anal fistula, as a means either of forestalling apprehended tuberculosis, or of arresting its progress when once established. The idea of averting or arresting tubercle necessarily involving the avoidance and abatement of every abnormal action and process, and of course, fistula in ano, *as such.*

It falls within the scope of the present paper, to allude to but one other class of objections that are raised against the course for which your committee is contending. It may be fitly characterized, perhaps, as the doctrine of substitution. Yielding the defence of fistula on the ground of its revellant or derivant agency, it still is claimed by some that in the case of fistulous discharge being tubercular in character, (a case which, if it exist, is regarded as quite *exceptional,*) it is unjustifiable to abate it, for the reason that the tubercular dyscrasy existing, it (the fistula) serves as a vent or safety-valve for its deposit and removal, and thus by vicarious action, preserving the more vital organs, the lungs, from its ravages.

Conceding, for the argument's sake, (what perhaps is more surmised than proved) that it assumes this vicarious office, and discharges tubercular matter, I would still insist that for the purposes of prophylaxis and of radical arrest, to which, be it remembered, our present knowledge summons and holds us, it is inadmissible, and that for the reason that it involves the fallacious and pernicious view, that our duty is met by furnishing an escape-pipe for disposing of the surplus deadly product, rather than eliminating the dyscrasy from the circulation and removing the conditions upon which it depends. Inadmissible, because it ignores the essential fact that our noble science and art, whilst it has demonstrated its feasibility, likewise calls us to the bold, aggressive attempts, of lifting up and holding up the animal and organic functions, *above the tubercle producing point.*

If at all admissible, it can only be in case of a palpably existing dyscrasy, and then, only as a very temporary and subsidiary expedient, to be laid aside at the earliest practicable moment, as being more harmful than hopeful, regard being had to the primary aim to which we address our efforts.

It remains only for your committee to indicate the case, or cases, in which alone, (if the process of reasoning here adopted is well founded and correct,) the cause of cherishing anal fistulæ is justified; and that, first, is in the somewhat mythical case just referred to, and for a merely temporary purpose.

The second, and only other case, that in the judgment of your committee furnishes an apology for its perpetration, is when the lungs have become so extensively involved as to furnish no rational hope either of arresting the morbid process, or (if unchecked) of long staving off a fatal result. When all hope has fled: when both attendant's and patient's "hearts are failing them for fear and for looking for what" is foreshadowed in the near future: when the question is narrowed down to one of a very little, or, a little more of time, and for any reason, a slight extension of the hold on life is desirable: it may be (especially in the case where the fistula takes on tubercular action) probably *is* indicated to cherish an existing fistula. For while, as we have essayed to show, it must be utterly impotent to avert or to arrest pulmonary tubercular deposition, it probably has on the vicarious principle, an extenuating agency, and may thus convert a lease of life, which, otherwise might be reckoned by weeks, into one of months, and it may be, years duration.

The question of advantage, then, to your committee's mind "hath this extent no more." And to promise to one's self, or to one's patient, in a case either of foreshadowed or well ascertained pulmonary tubercle, any hope of escape from a fatal termination, predicated exclusively, or mainly, upon the perpetuating of an anal fistula, is to insure a revenue of chagrin, and of sorrow, and perhaps of *reproach*, which is all the less to be coveted, because the mistake, though admitting of repentance and non-repetition, does not admit of reparation.

The truth, I fear, Mr. President and Gentlemen of the Association, must be confessed, that the most signal and honorable trophies which our science and art has achieved, or can yet achieve, in the conflict with this fell foe of our race, have been and are in the field of prophylaxis and prevention, and, perhaps, in the adjacent domain of accurately discerning and localizing incipient tubercle, and meeting it in its incipiency. As has been said, "peace has her victories no less than war," so may it be said, prophylaxis has her victories no less than therapeutics.

And as the true test of generalship consists, not so much in needless exposure of the lives of his braves in pitched battle with a superior foe, even though a costly victory is gained, as by finesse and forecast, in discovering, and by the acts of strategy and manœuvre, in eluding, and scattering, and wasting his resources of means and men. So is the merit of the medical man not infrequently to be tested: less by bold and heroic curative achievement, than by patient scrutiny and analysis of disease in its sources, nature, conditions, and relations, and the resulting ability to abate or to preclude its cause.

Thus when long and sad experience has taught us that we are impotent to stop, or greatly to control, the bitter waters when in full flow, we *may* do what is hardly less important, throw salt into the spring and purify the source, and so abate the cause.

It may, perhaps, be a more brilliant achievement in medicine to boldly attack, and, after a desperate hand to hand encounter, to conquer and expel some formidably intrenched disease; but it is a not less *real* triumph of our science and art, nor a less boon to our kind, to be able in a disease so widely prevalent and hitherto so inherently fatal, so truly an *opprobrium medicorum*, by our knowledge of its nature and conditions, to forestall and obviate it when inherited or apprehended.

This it now seems possible for legitimate medicine to do. Tuberculosis may, in a large majority of cases, it is believed, be avoided, if it cannot be cured. And never has the trite axiom, (with a variation,) "that an ounce of prevention is better than a pound of attempted cure," received a more perfect verification than is the case of our present knowledge of pulmonary tuberculosis.

Let this truth but be impressed upon the professional, and through it, upon the non-professional, mind, and medicine has achieved a triumph worthy of herself. This done, and we have conferred by far the most stupendous benefaction on humanity of which history furnishes any record. As one step in the right direction, *let anal fistulæ be ignored, exorcised, cast out.*

In the judgment of your committee, as has been already implied, the therapeutics of tuberculosis has lagged behind its etiology, pathology, and diagnosis. She is sadly out of harmony with her sister sciences, and there is an urgent indication that a work of reconstruction and readaptation should be brought out. Let her discordant elements be reattuned, and her parts readjusted, and then the utterance of her glorious symphony, whilst it gives glory and honor to medicine, shall be a jubilant pœan of hope and joy to long despairing, long suffering and terror struck humanity.

Your committee regrets that cases illustrative of his views have not yet fallen within the range of his observation, no cases sufficiently simple and uncomplicated to serve as types for the treatment contended for having occurred to him since attaining to full conviction of the truth of the present views. In one or two cases that were not quite of such character, the course advocated was recommended; but encountering the disapprobation of most medical men who were met with by my patients, it did not prevail.

Isolated cases, too, have been stated to your committee, that bore favorably upon the present views, but being matters of tradition and memory, rather than of precise diagnosis and record, they are now regarded as unfit to be adduced as testimony in the case.

In conclusion, your committee would express the wish that the positions and views so imperfectly taken, and the course so crudely advocated, shall meet with no favor from gentlemen of the association, except in so far as they are thought well founded upon a truthful basis; his object being not to dogmatise but to elicit discussion and find truth, the freest canvassing of his suggestions and arguments, would best comport with the object which impelled to the present report.

All of which is respectfully submitted.

*Prof. White* stated that in the earlier part of his practice, and when his attention was devoted to surgery, he had operated on a series of cases, some seven or eight of which he could recall to memory now, and which, so far as they went, would go to prove the policy of the operation.

Case I. A Mr. D., of light complexion, predisposed to tubercle, was operated on fifteen years since, and is still living.

II. A gentleman, about the same time, and under the same circumstances; still living.

III. Mr. B., died seven or eight years after the operation, of cirrhosis.

IV. A gentleman who was operated on twenty years since, died of pulmonary tubercle ten years after.

V. Another, a thin man, unhealthy, was operated on several years since, is now quite fleshy.

VI. A lady, who has always labored under consumptive symptoms, was operated on ten years ago. She has been more free from phthisical symptoms than before the operation.

VII. A gentleman now living, quite an old man, his family predisposed to consumption, was operated on ten years ago; is still living.

Prof. White added that in his opinion, it was proper to lessen the drain upon the system, where tubercular symptoms were present, by removing the anal affection.

*Prof. Flint* remarked that this was an important and interesting subject. The committee had argued the question ably, but to determine it we must have facts. It is doubtless true that many cases of anal fistula proceed from tubercular deposit in that region; but it is equally true that many cases are purely local and do not involve any general dyscrasis. In order to settle the question a distinction must be made between these two classes of cases. When the fistula is tubercular, we may take it for granted that tubercle exists also in the lung, in accordance with the law established by Louis. We must have facts, then, which would show what effect the operation had upon tubercular cases, excluding those purely local, about which there could be no dispute. A series of such cases would establish a law.

Prof. Flint stated further that he had recently observed some cases, which, so far as their number went, had some bearing on this discussion. One of these was a case of confirmed phthisis, complicated with anal fistula. It was remarkably prolonged in its duration. A second came to the hospital to be cured of anal fistula. He also had tubercle, and was advised to forego the operation, as it might possibly hasten the progress of disease in the lung which was then quiet. A third, with the same combination of diseases, has gained flesh and strength during the summer.

Here were three cases in which phthisis had progressed very slowly. Should this number be increased, and should it be found that anal fistula was a check upon the progress of the lung disease, it would follow that it should not be interfered with. On theoretical grounds he would agree with the committee. But it was possible that the anal fistula was a drain upon the blood dyscrasis, leading tubercle to deposit in that region instead of attacking the lungs.

*Prof. White* expressed great respect for the views of Prof. Flint, but, with our present views, we expect to arrest the deposit of tubercle by means invigorating the entire system. The drain of the anal fistula is an enfeebling agent, and in our general treatment just so much of the efficacy of remedies as was expended at the fistula, would be taken from the lung. Cases of prolonged tubercle, other than those associated with anal fistula, might be adduced. Was it not better to arrest tubercle wherever found, whether in the lungs or at the anus? The cases which he had cited would go to show that tubercle had been arrested, and life prolonged, by the operation

*Prof. Flint* replied that there seemed to be a misapprehension; he did not wish to be understood as opposing the views of Dr. Strong. His (Dr. Strong's) position was theoretically correct; to prove that it was practically so we must have facts. Again, he did not mean to say that fistula would act as a revulsive, but we now understand that tubercle involves a qualitative change in the blood, and that this expends itself at intervals in the deposit of successive crops of tubercles in the lungs. Assuming that such a crasis exists, is it not better that it should expend itself at the anus than in the lung? If this deposit accumulates and expends itself at the anus, had we not better treat it constitutionally and get rid of the tendency to deposit? If we find, then, that these cases are associated with slow progress of disease in the lung, we should look upon them as salutary. He would add another case: E. H. had tubercle fifteen years before death, which was cured. He finally had an anal fistula. This was cured by operation, but his phthisis returned, and he died shortly after.

*Prof. White* suggested that if the crop of anal tubercles remained dormant, we should be less likely to have a new crop than if suppuration occurred. Suppuration, by exhausting the system, increases the chances of new deposition.

*Prof. Rochester* was much pleased with the argument of Dr. Strong, but thought that we might, perhaps, be going too far, even in denying a revulsive action to anal tubercle. All of us, though having long abandoned depletion in the cure of phthisis, must have had opportunities of observing the salutary effect of a seton on the arm in retarding its progress. He would, however, state a case which went in favor of the views of the committee. A young woman, of strong hereditary tendency, had tubercle, which was very rapid in its progress. Four months before death, she had anal fistula opening in one of the labia, which was more troublesome, and to all appearance, more exhausting than the lung disease. She died suddenly from perforation of the pleura, producing pneumo-hydro-thorax.

*Dr. Ham* thought that we might get some light by reasoning from analogy. In critical discharges, such as hæmorrhage in typhoid fever even, we sometimes saw great benefit resulting from the hæmorrhage, though no one would think of venesection in such a case. Might not fistula also have a similar action?

*Dr. Wyckoff* mentioned a case of anal fistula, where the lungs were, to all appearance, sound. No examination of them was made, but the patient had no cough and appeared healthy. He was operated on for the fistula, and died a few months after from phthisis. He inquired how we should be able to distinguish when the fistula was tuberculous.

*Prof. Flint* replied that we must learn from dyscrasic appearances, and the general rule of Louis.

*Dr. Van Pelt* said that as it was necessary to draw a line of distinction between cases, tubercular or non-tubercular, the microscope would assist in making the diagnosis. We might try a general treatment first, and continue it up to the point where an operation would be safe.

*Dr. Nott* reported a case of rupture of the liver, the second within a few months. A child, aged six years, was caught under a wagon wheel, and slid along on the icy street for some distance. The wheel did not pass over it. It died half an hour afterward. There were no external marks. On opening the abdomen it was found filled with fluid blood; bowels normal; and the right lobe of the liver broken in two.

On the day previous he had held a post-mortem examination on the body of a woman, 27 years of age, who had an infant, seven months old. The woman was found dying in her bed in the morning. Had had ague five years previously; had been subject to nightmare. On examination, the liver was found weighing five pounds, and pushed up very high in the right hypochondrium, so as to encroach much upon the right pleural cavity. The spleen weighed twenty ounces. The heart was elevated in the chest so that the apex rested under the left nipple. The upper lobe of left lung was emphysematous; lower down was an apoplectic clot.

On inquiry, by the president, scarlatina was reported as prevalent, but not in a malignant form. Its treatment was discussed at some length, but nothing of importance was adduced.

In reference to the prophylaxis of scarlatina, Drs. Van Pelt, White, Flint, and Rochester, had used belladonna, but had abandoned it as entirely useless.

And the association then adjourned.

SANFORD B. HUNT, Secretary.

